# A benchmark dataset for machine learning–based prediction of hydrogen evolution efficiency from GLFO-catalyzed sucrose photocatalysis

**DOI:** 10.1016/j.dib.2026.112996

**Published:** 2026-06-20

**Authors:** Rezan Bakır, Ceren Orak, Halit Bakır

**Affiliations:** a Department of Computer Engineering, Faculty of Engineering, Sivas University of Science and Technology, Sivas, Turkey; b Department of Chemical Engineering, Faculty of Engineering, Sivas University of Science and Technology, Sivas, Turkey

**Keywords:** Hydrogen production, Photocatalysis, Artificial intelligence, Dataset

## Abstract

This study introduces a novel experimental dataset designed to support the development and evaluation of artificial intelligence models for photocatalytic hydrogen production. The dataset was generated from a series of controlled laboratory experiments utilizing a graphene-supported LaFeO₃ (GLFO) catalyst and sucrose solution as the organic sacrificial agent. Key process variables—including solution pH, catalyst concentration, and initial hydrogen peroxide concentration (HPC)—were systematically varied using a Box–Behnken design to investigate their influence on hydrogen evolution. A total of 1370 data points were collected, each representing a unique combination of input parameters and the corresponding hydrogen yield (mmol gcat⁻¹). The dataset provides high-resolution insight into nonlinear interactions in the hydrogen generation process and is intended as a benchmark resource for reproducible research in machine learning-driven photocatalysis. Initial analysis confirms the dataset’s suitability for training predictive models and evaluating optimization strategies. By releasing this dataset, we aim to advance reproducible research and accelerate the application of AI in clean energy technologies, particularly in the field of photocatalytic hydrogen production.

## Graphical abstract

**Figure:**
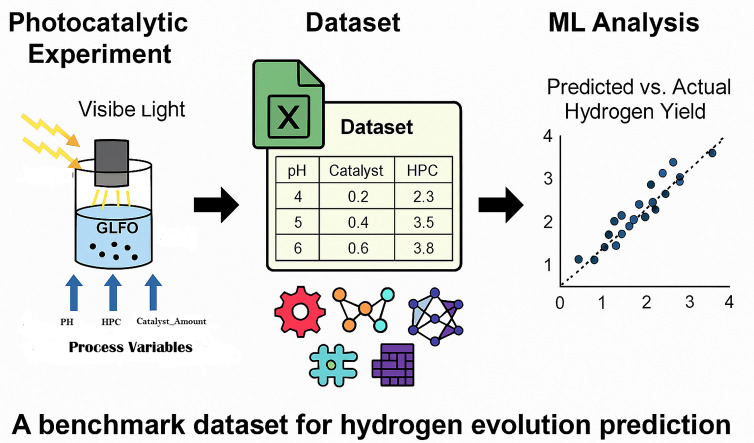

Specifications Table

This table provides a high-level technical overview of your dataset.

**Table:** 

Subject	Chemical Engineering (general) / Computer Science (Artificial Intelligence)

Specific subject area	photocatalytic hydrogen production modeling and machine learning benchmarking.
Type of data	tables (Excel) and figures (heatmaps, 3D surface plots, and importance charts).
Data collection	controlled laboratory experiments using a batch photocatalytic reactor equipped with gas chromatography (TCD detector).
Data format	raw experimental measurements and pre-processed (normalized) data for ML training.
Description of data collection	1370 data points were generated using a Box–Behnken experimental design. The process involved varying solution pH, catalyst concentration, and hydrogen peroxide concentration.
Data source location	Sivas University of Science and Technology, Sivas, Turkey.
Data accessibility	Repository name: Kaggle. Direct link: https://www.kaggle.com/datasets/razanghanem/machine-learning-dataset-for-hydrogen-evolution

## 1 Value of the Data

• **Pioneering resource for AI in hydrogen energy**: this study provides the first comprehensive experimental dataset specifically designed for the development and evaluation of machine learning (ML) and deep learning (DL) models in the domain of photocatalytic hydrogen production using graphene-supported lanthanum ferrite (GLFO).
• **High-resolution benchmarking**: with 1370 high-resolution data points, the dataset serves as a critical benchmark for testing the predictive accuracy and generalization of diverse regression algorithms, ranging from linear models to advanced ensemble-based approaches.
• **Optimization of nonlinear interplay**: the use of a Box–Behnken experimental design allows researchers to explore the complex, nonlinear interactions between process variables such as pH, catalyst concentration, and hydrogen peroxide concentration, which are otherwise difficult to capture through traditional empirical methods.
• **Support for reproducible research**: by releasing a pre-cleaned, validated, and structured dataset free from missing values, this work fosters transparency and reproducibility within the green hydrogen research community.
• **Industrial and environmental relevance**: the dataset models hydrogen evolution from sucrose solutions, effectively simulating the treatment of sugar industry wastewater and providing data-driven insights for circular economy and waste-to-energy applications.
• **Compatible for advanced modeling**: the structured numeric format of the data ensures immediate compatibility with major AI frameworks such as Scikit-learn, TensorFlow, and PyTorch, facilitating the development of automated modeling workflows and physics-informed machine learning.

## 2 Background

Photocatalytic hydrogen production has garnered considerable interest as a sustainable pathway toward a carbon-neutral energy economy. Among various semiconductor materials explored, LaFeO₃, a perovskite oxide, and its graphene-supported derivatives have shown promising photocatalytic activity under visible light due to their tunable band structure, chemical stability, and enhanced charge transport properties [1]. However, optimizing reaction parameters such as catalyst concentration, pH, and hydrogen peroxide concentration is complex, requiring significant experimental effort. Recent studies address this bottleneck through ML, which has emerged as a robust tool for data-driven prediction and optimization in photocatalytic systems [2]. Recent advances in photocatalytic materials have significantly enhanced the prospects of solar-driven hydrogen production and environmental remediation. Among these materials, graphitic carbon nitride (g-C₃N₄)-based photocatalysts have attracted considerable attention due to their visible-light activity, chemical stability, and cost-effectiveness. Various modification strategies, including heterojunction engineering, cocatalyst incorporation, and composite formation, have been developed to improve photocatalytic performance. Recent studies have demonstrated the effectiveness of g-C₃N₄-based systems in hydrogen generation, photocatalytic reduction, and pollutant degradation applications [[3], [4], [5]]. These developments underscore the growing need for high-quality experimental datasets to facilitate data-driven modeling and optimization of photocatalytic processes.

Our study contributes to this evolving landscape by releasing a comprehensive dataset derived from laboratory experiments using a graphene-supported LaFeO₃ (GLFO) photocatalyst with sucrose as the organic sacrificial agent. Using a Box–Behnken design, we explored the nonlinear interplay of reaction variables and their effect on hydrogen yield, resulting in a dataset of 1370 high-resolution data points. This dataset can serve as a benchmark for machine learning and deep learning applications in the domain of solar-driven hydrogen evolution.

The potential of ML in modeling photocatalytic systems has already been demonstrated across various catalyst families. For instance, authors in [6] study applied ensemble models—random forest, lightGBM, and bagging regressor—for predicting hydrogen evolution using LaFeO₃-based materials. Similarly, in their study, Bakır et al. [7] utilized a stacked learning framework on sugar-industry wastewater and GLFO catalysts, confirming the robustness of ensemble approaches. Other efforts combined experimental and ML strategies, such as the work by Arabacı et al. [8], who integrated experimental insights with ML to predict hydrogen output from Fe/g-C₃N₄ photocatalysts.

Further advancements include studies on Cu/g-C₃N₄ systems [9], where regression algorithms outperformed traditional statistical tools in accuracy and generalization. Additionally, researchers have explored multi-component hybrid catalysts and bio-derived materials using ML for hydrogen evolution predictions [10]. These models often integrate feature engineering, ensemble stacking, or hybrid neural networks, achieving high predictive performance with minimal experimental data. In this context, our dataset fills a critical gap by offering a large, diverse, and well-curated resource focused on a visible-light-active GLFO catalyst system. It supports reproducible research and the development of universal predictive models that can be generalized across catalyst types and reaction environments.

The dataset is accompanied by a comprehensive Kaggle repository. The inclusion of multiple learning models and visualizations provides both a reference point and a starting framework for future research in the optimization of hydrogen production systems. By making this dataset publicly available, we aim to foster reproducibility, comparability, and accelerated development in the photocatalysis and green hydrogen research communities.

## 3 Data Description

The dataset consists of a single structured file containing 1370 unique records. Each record represents a 4-hour experimental run with the following columns:

• **pH**: the acidity/alkalinity of the reaction solution, ranging from 2 to 11.
• **Catalyst amount (g/L)**: the concentration of the graphene-supported LaFeO₃ (GLFO) catalyst (0.05 to 0.4 g/L).
• **HPC (mM)**: the initial concentration of hydrogen peroxide (0 to 40 mM).
• **Hydrogen yield (mmol gcat⁻¹)**: the primary output variable representing the measured efficiency of hydrogen evolution.

## 4 Experimental Design, Materials, and Methods

### 4.1 Experimental materials

GLFO catalyst was synthesized using the sol-gel method and employed as the photocatalyst in all experiments. Analytical-grade sucrose was used as the organic sacrificial agent. Hydrogen peroxide (H_2_O_2_) solution (30%w/w) was used to investigate its effect on the photocatalytic reaction. All chemicals were used without further purification.

### 4.2 Experimental setup

The experiments were conducted in a cylindrical glass reactor with a total volume of 1 L containing 500 mL of reaction solution. Illumination was provided by a compact fluorescent lamp (Philips Master PL-L, 24 W) with a luminous flux of 1800 lm and an approximate light intensity of 435 W m-2. The reaction temperature was maintained at 25 °C throughout the experiments. Hydrogen evolution was analyzed using a gas chromatograph (Agilent 6890) equipped with a thermal conductivity detector (TCD). A Molecular sieve 5A column was employed, using N2 as the carrier gas at a flow rate of 30 mL min-1, while the oven temperature was maintained at 30 °C during analysis. Hydrogen production was monitored continuously over a 4-hour reaction period. The low and high values of key reaction parameters were listed in Table 1.

**Table 1:** Experimental range of key reaction parameters.

Parameter	Minimum	Maximum
pH	2	11
Catalyst amount (g/L)	0.05	0.4
Hydrogen peroxide concentration (mM)	0	40

### 4.3 Experimental design and variable range

A Box–Behnken design (BBD) was employed to efficiently explore the experimental space while minimizing the number of trials. The output variable for each experimental run was the hydrogen yield (mmol gcat⁻¹).The following three input variables were selected based on their known influence on hydrogen evolution:

Among these variables, HPC was included because of its significant influence on photocatalytic charge-transfer processes. H_2_O_2_ can act as an electron acceptor, reducing electron-hole recombination and enhancing charge separation efficiency. Furthermore, H_2_O_2_ may participate in the consumption of photogenerated holes and the generation of reactive oxygen species, thereby affecting the hydrogen evolution pathway. Depending on its concentration, H_2_O_2_ may either enhance hydrogen production through improved charge carrier management or suppress it by competing with proton reduction reactions and consuming photogenerated electrons. Therefore, HPC was selected as an independent variable to capture its nonlinear effect on hydrogen evolution performance.

### 4.4 Sample records

A few example records from the dataset are shown in Table 2. These records demonstrate how changes in experimental conditions lead to variations in hydrogen output, offering an excellent foundation for predictive modeling.

**Table 2:** Selected records from the collected data.

Reaction conditions			Produced Hydrogen (mmol gcat⁻¹)
pH	catalyst amount (g/L)	HPC (mM)	
2.5	0.1	0	3.24
2	0.1	10	3.05
2.3	0.1	10	3.06
3	0.4	10	2.94
2.8	0.1	20	2.92
5.5	0.1	20	3.27
7.4	0.1	20	3.52
4.6	0.2	30	2.73
7.7	0.2	30	3.21
2.9	0.3	40	1.73
4.5	0.3	40	1.89
8.2	0.3	40	2.25
2	0.1	15	2.70
9	0.1	15	4.01
8	0.1	25	4.26
4	0.1	35	3.09
5	0.2	5	2.98
4	0.05	25	3.32
9	0.15	5	3.31
9	0.35	35	3.34
9	0.4	35	3.48

### 4.5 Data preprocessing

Prior to applying machine learning and deep learning models, several preprocessing steps were performed to ensure the quality and consistency of the dataset. These steps were essential for eliminating bias, improving training stability, and enabling accurate model predictions.

#### 4.5.1 Normalization

All input features—pH, catalyst amount (g/L), and hydrogen peroxide concentration (HPC, mM)—were scaled using the MinMaxScaler method, which maps each feature to the [0, 1] interval. This transformation improves numerical stability and can facilitate model training and convergence.The output variable, hydrogen yield (mmol gcat⁻¹), was left unscaled to retain its original interpretability.

#### 4.5.2 Dataset splitting

The dataset, consisting of 1370 unique samples, was randomly divided into:

• **Training set**: 80% of the data, used for model learning.
• **Test set**: 20% of the data, reserved for evaluating generalization.

This random partitioning ensured that both subsets represented the full variability of the experimental data.

#### 4.5.3 Missing value handling

No missing or null values were observed in the dataset due to the systematic and controlled nature of the experimental design. Thus, no imputation procedures were necessary.

#### 4.5.4 Data consistency and validation

Prior to training, each feature was visually and statistically inspected for outliers and abnormal distributions. Due to the use of a Box–Behnken experimental design, the data exhibited a uniform and structured spread across the feature space. No extreme outliers were detected that required removal or transformation.

### 4.6 Feature selection

As the dataset was generated from a controlled process involving a small number of experimentally validated features, no dimensionality reduction or additional feature selection methods were applied. The three input variables were retained in their original form for modeling purposes.

## 5 Machine Learning Benchmarking

To verify the reliability and usability of the dataset for AI-driven modeling tasks, multiple machine learning algorithms were applied to predict hydrogen evolution efficiency based on the three experimental input features: pH, catalyst concentration, and hydrogen peroxide concentration. The dataset was randomly divided into training (80%) and testing (20%) subsets, and each model was evaluated using standard regression metrics, including mean absolute error (MAE), root mean squared error (RMSE), and the coefficient of determination (R²).

A diverse set of models was tested (see Table 3), including linear regression, support vector regression (SVR), decision tree, random forest, gradient boosting, K-nearest neighbors (KNN), bagging, adaboost, extra trees, and a basic multi-layer perceptron (MLP). All benchmark models were implemented using the default hyperparameter settings provided by the Scikit-learn library.

**Table 3:** Benchmarking machine learning algorithms using the proposed dataset.

Model	MAE	RMSE	R²
Linear regression	0.2935	0.3985	0.1509
Random forest	0.0179	0.0545	0.9841
Gradient boosting	0.0797	0.1389	0.8969
Support vector regressor	0.1322	0.2407	0.6900
Decision tree	0.0269	0.0710	0.9730
KNN regressor	0.0418	0.1163	0.9276
AdaBoost regressor	0.1791	0.2299	0.7173
Bagging regressor	0.0180	0.0549	0.9839
Extra trees regressor	0.0127	0.0473	0.9880
MLP regressor	0.1331	0.2152	0.7524

The benchmarking analysis was intended to provide a baseline assessment of dataset usability rather than a comprehensive model optimization study. Since the dataset was generated using a Box–Behnken experimental design, which may produce structured response surfaces, the reported results should be interpreted as an initial demonstration of predictive potential rather than definitive evidence of model generalization. Accordingly, users are encouraged to employ more rigorous validation strategies, including k-fold cross-validation, repeated cross-validation, hyperparameter optimization, and independent external validation datasets when developing predictive models based on this benchmark resource.

The ensemble-based models, particularly the extra trees regressor (R² = 0.9880) and random forest regressor (R² = 0.9841), demonstrated superior predictive accuracy, confirming the dataset’s high quality and suitability for data-driven modeling. These results indicate strong internal consistency and a well-structured relationship between input features and output targets, thereby validating the dataset for further machine learning and deep learning experimentation.

It is also worth noting that this dataset has been utilized in several subsequent studies conducted by the authors, where more rigorous validation frameworks, including k-fold cross-validation, repeated random-seed validation, hyperparameter optimization, and overfitting mitigation strategies, were employed. These studies consistently reported strong predictive performance and stable generalization behavior, providing additional evidence for the reliability and utility of the dataset in machine learning-based hydrogen production modeling.

### 5.1 Usage notes

This dataset is intended to support the development and evaluation of AI-based models—particularly those focused on regression tasks in the domain of photocatalytic hydrogen production. Researchers can use this dataset to:

• Train and benchmark machine learning or deep learning models for predicting hydrogen yield.
• Perform feature selection or sensitivity analysis to determine which experimental parameters most influence hydrogen evolution.
• Explore automated modeling workflows using hyperparameter tuning, cross-validation, and ensemble methods.
• Develop physics-informed ML models by combining domain knowledge with data-driven approaches.

Due to the numeric and structured nature of all input and output features, the dataset is directly compatible with most ML/DL frameworks such as Scikit-learn, TensorFlow, PyTorch, and XGBoost. The dataset is free of missing values, pre-cleaned, and ready for use in supervised learning pipelines.

### 5.2 Data availability statement

The complete dataset, including the experimental input variables (pH, catalyst concentration, and hydrogen peroxide concentration) and the corresponding hydrogen evolution measurements, is publicly available on Kaggle. The dataset is provided in Excel (.xlsx) format and includes a separate readme file that explains the structure, units, and meaning of each column. Researchers can directly load the dataset into Python-based environments such as Scikit-learn, TensorFlow, or PyTorch for training and testing AI models. The dataset is hosted at: https://www.kaggle.com/datasets/razanghanem/machine-learning-dataset-for-hydrogen-evolution.

### 5.3 Suggested visualizations

**Fig. 1:**
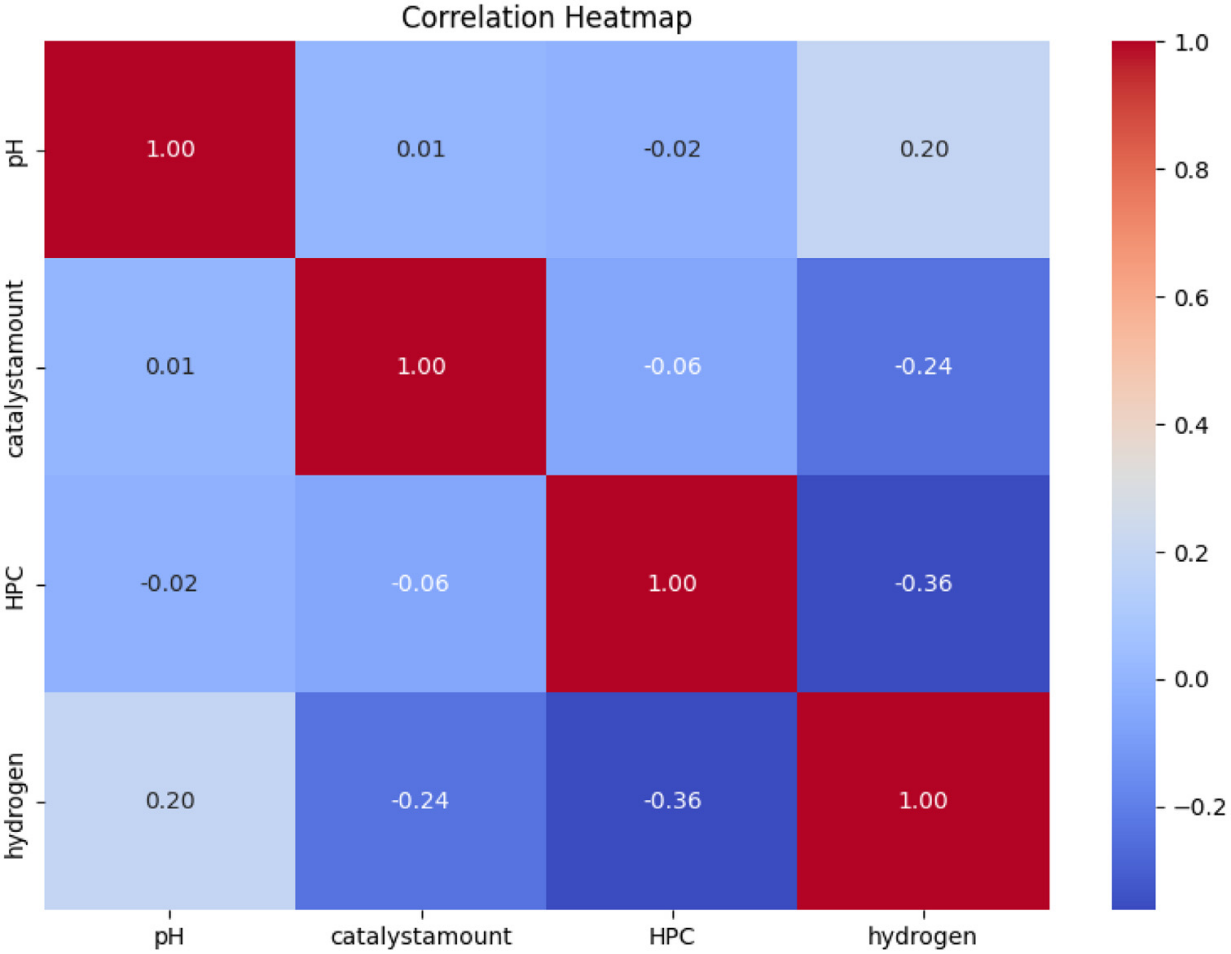
Correlation heatmap illustrating the relationships among the experimental input variables (pH, catalyst concentration, and hydrogen peroxide concentration) and the output variable (hydrogen yield). The heatmap provides a visual overview of the strength and direction of pairwise correlations within the dataset and highlights the influence of individual process parameters on hydrogen production performance.

**Fig. 2:**
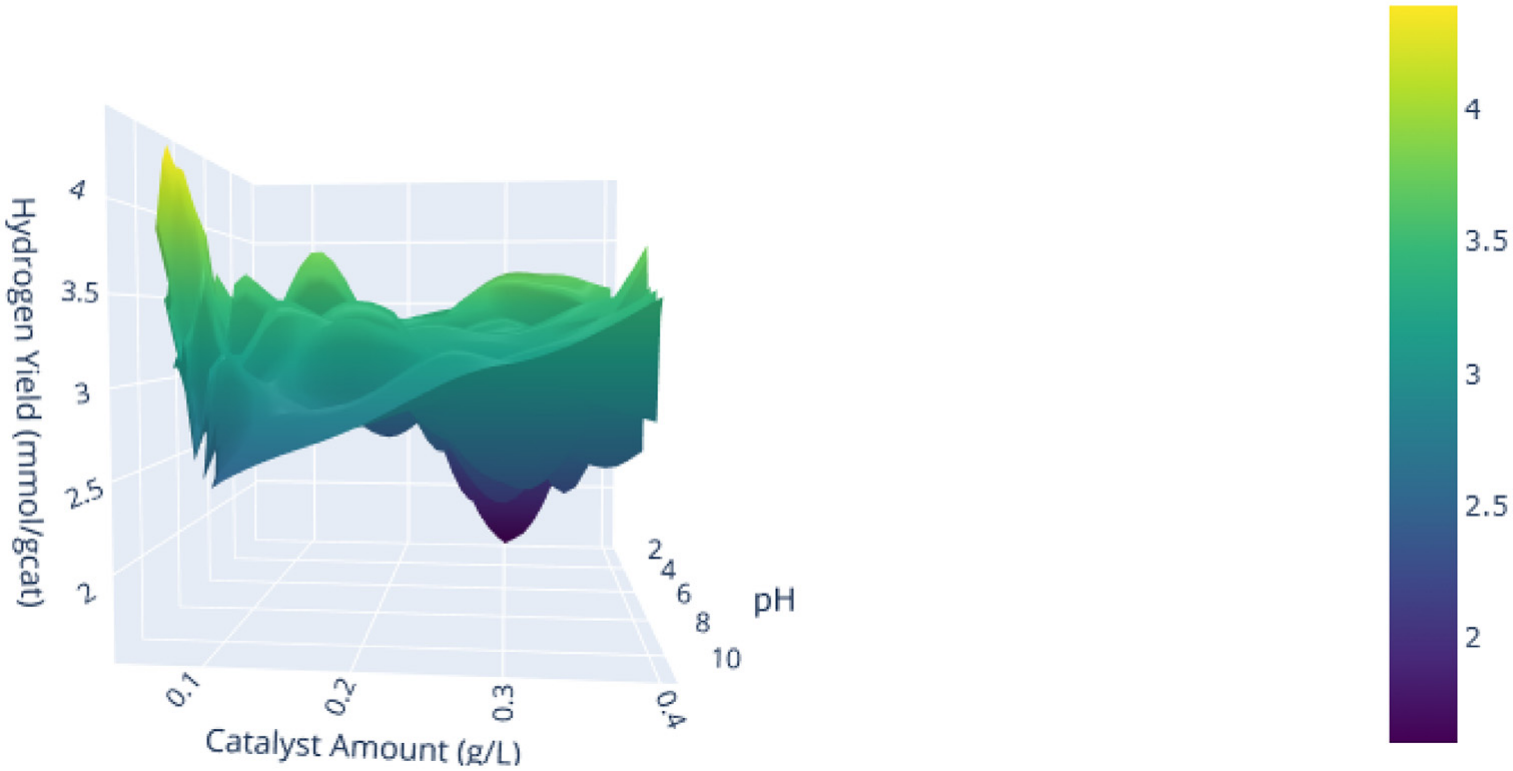
Hydrogen yield versus pH and catalyst amount. Three-dimensional surface plot showing the variation of hydrogen yield as a function of solution pH and catalyst concentration. The figure illustrates the nonlinear interaction between these two process variables and their combined effect on photocatalytic hydrogen evolution efficiency under GLFO-catalyzed conditions.

**Fig. 3:**
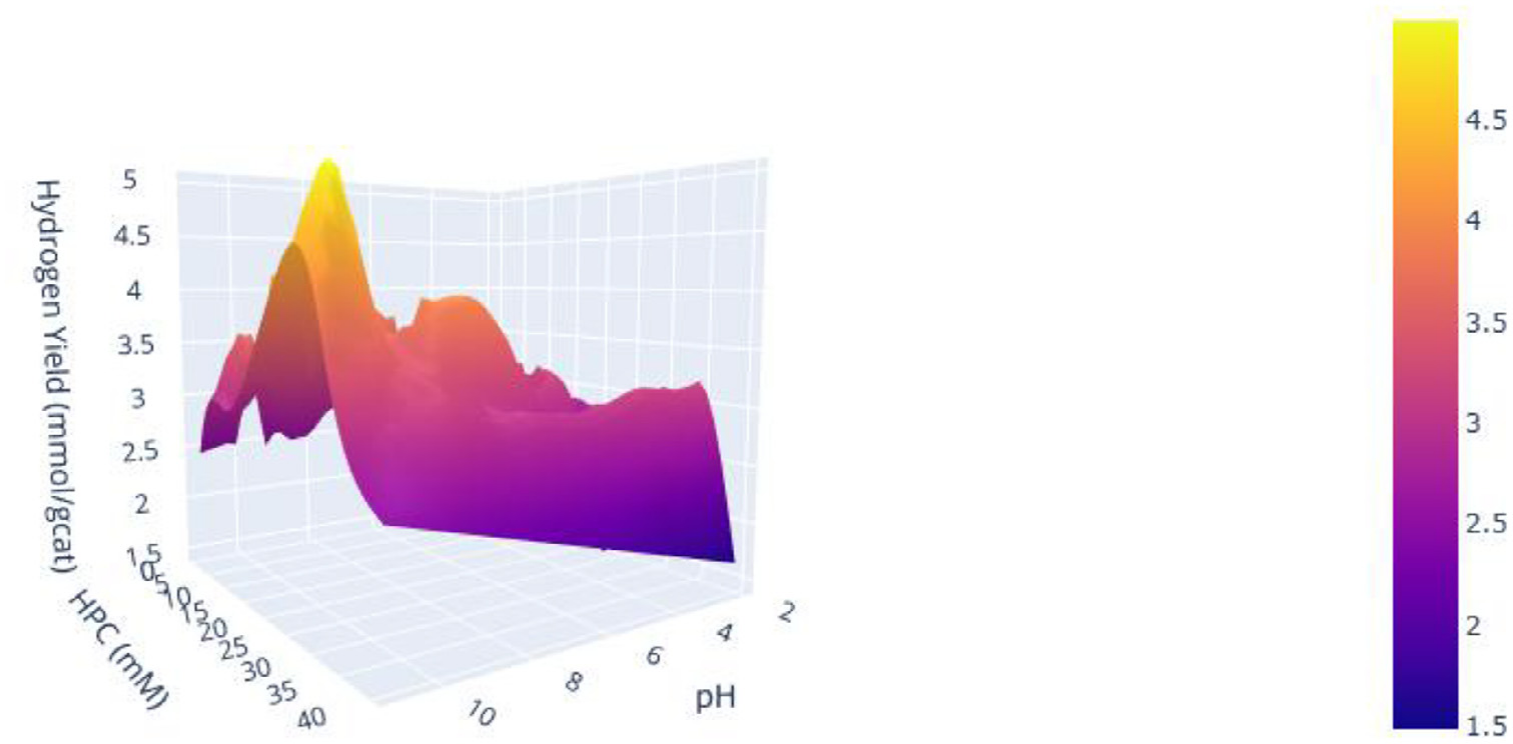
Hydrogen yield versus pH and hydrogen peroxide concentration. Three-dimensional surface plot demonstrating the dependence of hydrogen yield on solution pH and hydrogen peroxide concentration (HPC). The visualization highlights the complex response behavior of the photocatalytic system and the influence of oxidant concentration across different pH conditions.

**Fig. 4:**
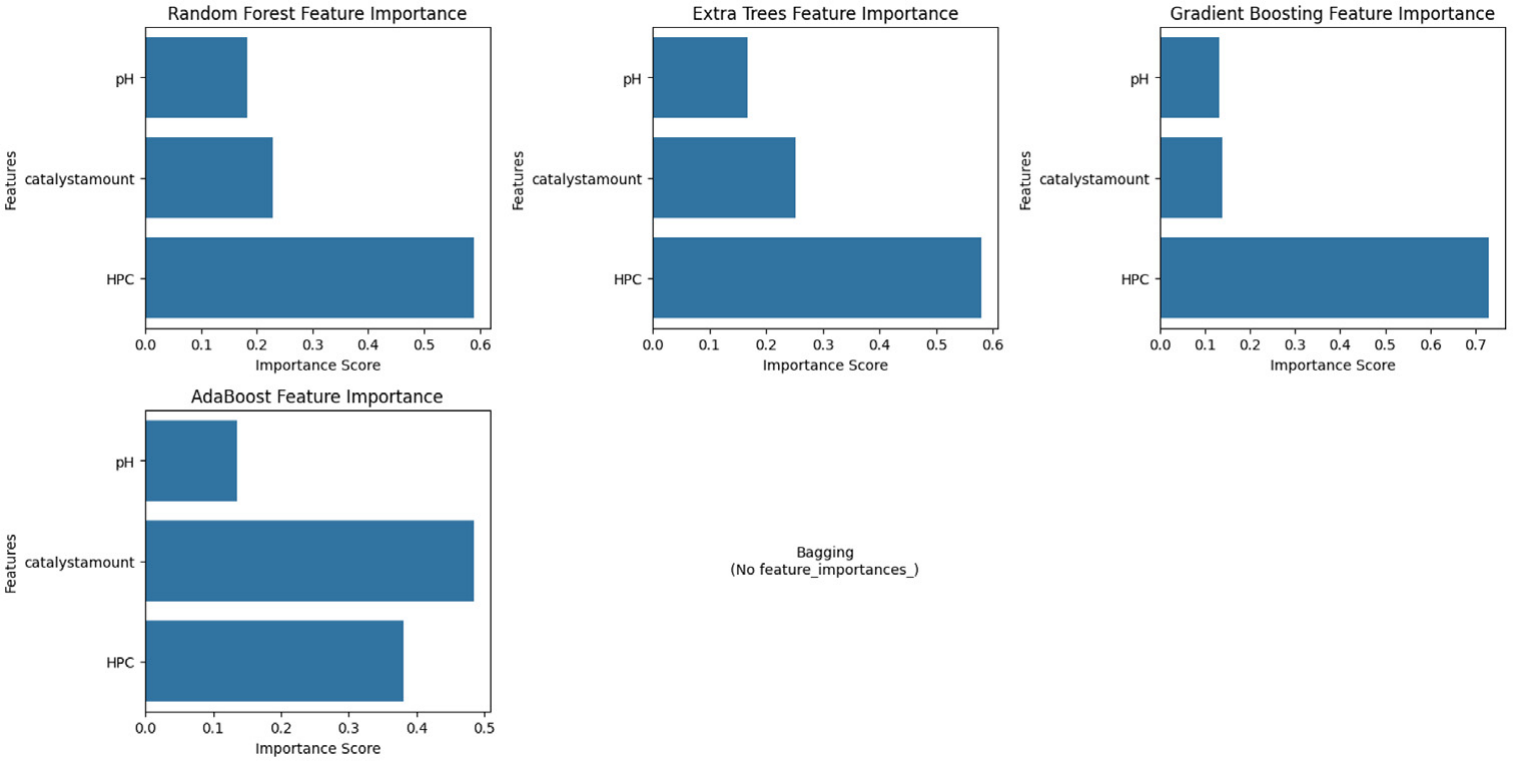
Feature importance scores obtained from ensemble-based machine learning models, including random forest and extra trees regressors. The figure compares the relative contribution of pH, catalyst concentration, and hydrogen peroxide concentration to the prediction of hydrogen yield, providing insight into the most influential variables in the dataset.

To better understand data trends and model behavior, the following plots (Fig. 1, Fig. 2, Fig. 3, Fig. 4) are recommended and provided:

• **Correlation heatmap** showing relationships between pH, catalyst concentration, hydrogen peroxide concentration, and hydrogen output.
• **3D surface plots** visualizing hydrogen yield as a function of two input parameters, such as (pH, catalyst amount).
• **Feature importance bar chart** from ensemble models such as Random Forest or Extra Trees, indicating the influence of each input variable on hydrogen prediction.

## Limitations and Future Works

While the dataset captures the relationship between three experimental parameters and hydrogen evolution, it does not account for factors such as reaction temperature, illumination intensity, or catalyst particle size. These limitations suggest opportunities for expanding the dataset to support more comprehensive modeling in future studies.

## CRediT Author Statement

**Rezan Bakır:** conceptualization, methodology, software, validation, formal analysis, investigation, data curation, writing - original draft, visualization, supervision. **Ceren Orak:** methodology,data collection, investigation, resources, writing - original draft, writing - review & editing. **Halit Bakır:** conceptualization, methodology, software, validation, writing - original draft, writing - review & editing.

## Ethics Statement

The authors have read and followed the ethical requirements for publication in Data in Brief and confirm that the current work does not involve human subjects, animal experiments, or any data collected from social media platforms.

## Declaration of Generative AI and AI-assisted Technologies in the Writing Process

The authors used OpenAI's ChatGPT to assist solely with language correction and grammar improvements during the preparation of this manuscript. All content was created and reviewed by the authors.

## Funding

This work was supported by the Scientific and Technological Research Council of Turkey (TÜBİTAK) under Project No. 224M008.

## Data Availability

kaggleMachine Learning Dataset for Hydrogen Evolution (Original data).
